# Hydrologic Evaluation of TRMM Multisatellite Precipitation Analysis for Nanliu River Basin in Humid Southwestern China

**DOI:** 10.1038/s41598-017-02704-1

**Published:** 2017-06-01

**Authors:** Yinjun Zhao, Qiongying Xie, Yuan Lu, Baoqing Hu

**Affiliations:** 1 Key Laboratory of Environment Change and Resources Use in Beibu Gulf, Ministry of Education, Guangxi Teachers Education University, 175 Mingxiu East St., Nanning, 530001 China; 2 School of Geography and Planning, Guangxi Teachers Education University, 175 Mingxiu East St., Nanning, 530001 China

## Abstract

The accuracy of Tropical Rainfall Measuring Mission (TRMM) multi-satellite precipitation analysis (TMPA) daily accumulated precipitation products (3B42RTV7 and 3B42V7) was evaluated for a small basin (the Nanliu river basin). A direct comparison was performed against gauge observations from a period of 9 years (2000–2009) at temporal and spatial scales. The results show that the temporal-spatial precipitation characteristics of the Nanliu river basin are highly consistent with 3B42V7 relative to 3B42RTV7, with higher correlation coefficient (CC) approximately 0.9 at all temporal scales except for the daily scale and a lower relative bias percentage. 3B42V7 slightly overestimates precipitation at all temporal scales except the yearly scale; it slightly underestimates the precipitation at the daily spatial scale. The results also reveal that the precision of TMPA products increases with longer time-aggregated data, and the detection capability of daily TMPA precipitation products are enhanced by augmentation with daily precipitation rates. In addition, daily TMPA products were input into the Xin’anjiang hydrologic model; the results show that 3B42V7-based simulated outputs were well in line with actual stream flow observations, with a high CC (0.90) and Nash-Sutcliffe efficiency coefficient (NSE, 0.79), and the results adequately captured the pattern of the observed flow curve.

## Introduction

Precipitation is a complex natural event that exhibits significant variability at both temporal and spatial scales. Most complex hydrological phenomena occur due to its temporal and spatial variability, and therefore, highly accurate temporal-spatial and long term precipitation data are essential for research on or predications of climate change, for studies involving hydrologic simulation and prediction, and for natural hazards and water resource management. Point rainfall measurement at rain gauge stations, ground-based weather radar observation, and satellite-based precipitation data collection using remote sensing technology are the three main sources of rain data. Among these, ground-based rain gauges provide the most accurate measurements of precipitation, but ground-based rain gauge networks are often unevenly distributed in a given area due to climatic, economic and other limiting conditions. Furthermore, rain gauge data (both temporal and spatial) are either sparse or non-existent for many parts of the world, particularly in developing countries^1^. It is not advantageous to rely on precipitation measured by only a certain number of rain gauges as being representative^2^. Regarding weather radar-based measurement of precipitation, coverage is often not dense enough over most parts of the world. In general, satellite-based precipitation products have several advantages over ground-based observation, such as easy data acquisition, large coverage, appropriate temporal and spatial resolutions, long-term and continuous recording, and fewer impacts from climate and terrain variability. Thus, satellite-based technologies have witnessed significant development in recent years. Various high-resolution satellite quantitative precipitation estimation (QPE) products have been developed, e.g., Tropical Rainfall Measuring Mission (TRMM), Global Satellite Mapping of Precipitation (GSMap), Global Precipitation Climatology Project (GPCP), Climate Prediction Center (CFC) morphing method (CMORPH), and Precipitation Estimation from Remotely Sensed Information using Artificial Neural Networks (PERSIANN).

TRMM is one of the most important precipitation projects currently in operation, with widespread coverage (50°S~50°N). The National Aeronautics and Space Administration (NASA) Goddard Space Flight Center (GSFC) developed TRMM Multi-satellite Precipitation Analysis (TMPA) products to provide near-real-time (TMPA 3B42RTV7, hereafter referred to as 3B42RTV7) and post-real-time (TMPA 3B42V7, hereafter referred to as 3B42V7) research versions of precipitation data.

3B42V7 is the latest version, replacing 3B42V6 as of 2011. The errors and uncertainties of these QPE products greatly impact their applications and hence the application results^3^. TMPA data have been broadly evaluated^1, 4–8^ and employed for numerous applications such as hydrological modeling^3, 9^, soil moisture predication^10^, fire detection^11^, and agriculture^12^. The accuracy of TRMM products has been accepted for most low-latitude areas and large-medium scales, but few studies have reported evaluations of the products against rain gauge observations for small basin areas or the Beibu Gulf region.

**Figure 1:**
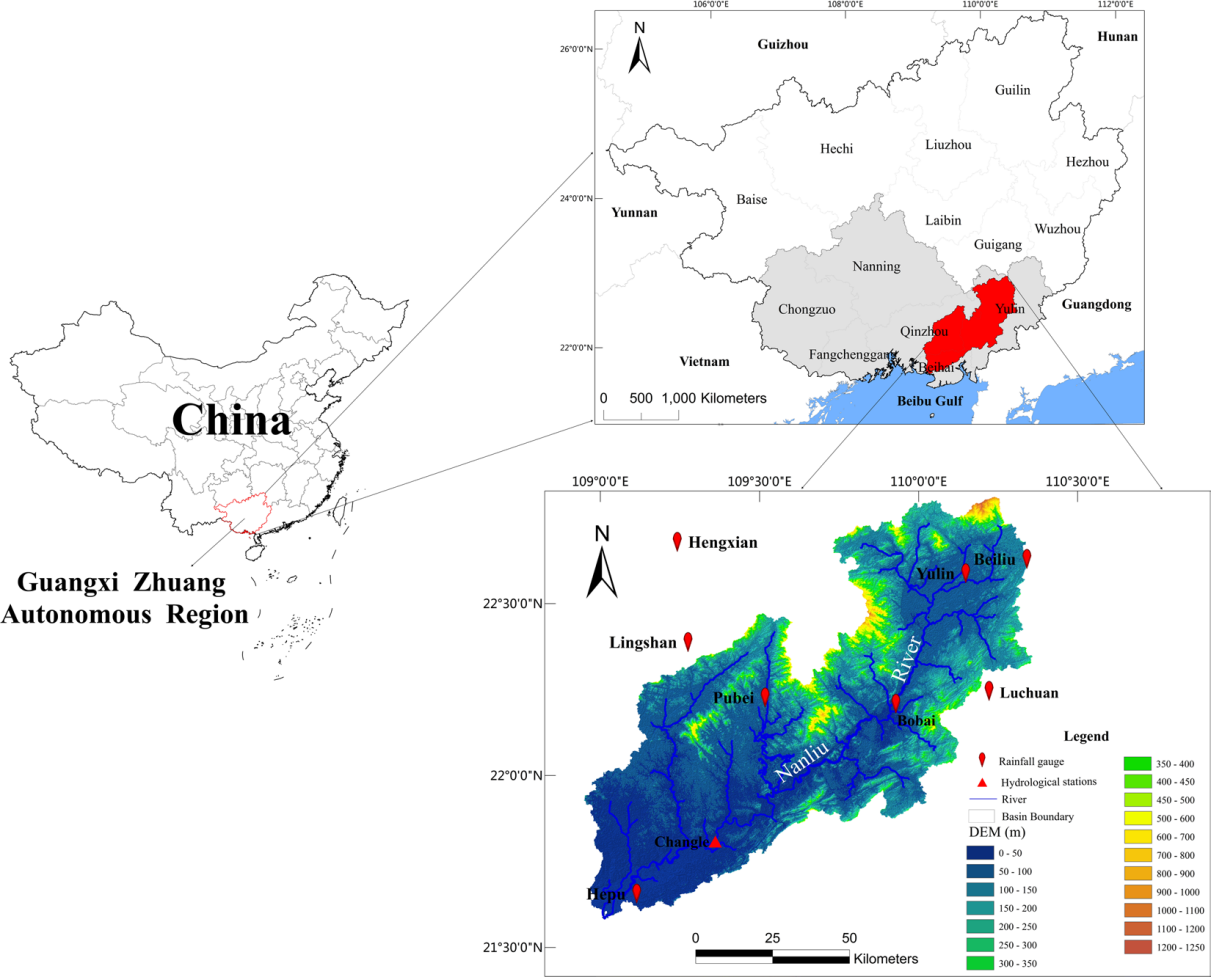
The location and range of the study area (Nanliu river basin) along with corresponding data, generated by ArcGIS10.1 (http://www.esrichina.com.cn/softwareproduct/ArcGIS/). Seven rainfall gauges located in the Nanliu river basin were selected as reference stations for data to compare with TMPA products. The gauges are evenly distributed over the Nanliu river basin and are manually maintained and managed. These gauges provided high quality observation data for the period 2000–2009 and weren’t transmitted on the international circuit of the Global Telecommunication System (GTS); data were collected from the Meteorological Service of Guangxi. The Changle hydrologic station is the main station in the region and has been providing long-term measurements since it was built in 1952. The station is located at the south end of the Nanliu river basin and controls a drainage area of 6592 km^2^ that accounts for 72.5% of the total basin area. Its mean annual runoff is approximately 56.1 × 10^9^ m^3^, with 81% of the runoff occurring during the flood season. Data on the daily stream flow and evaporation from January 2007 to March 2009 were collected from hydrologic yearbooks at Changle station. High resolution in spatial (0.25°) and temporal (daily) datasets were obtained from the National Aeronautics and Space Administration (NASA) archive (http://disc.gsfc.nasa.gov/) for two standard TMPA products, 3B42RTV7^13^ and 3B42V7^14^ (bias-adjusted by monthly rain gauge data), for the time period 2000~2009. The daily accumulated precipitation product is generated from the research-quality 3-hourly TMPA (3B42) at the NASA GES DISC as a value added product. The daily data were then aggregated into monthly, seasonal, flood/non-flood seasonal and annual precipitation datasets. The four seasons used at for the seasonal scale were denoted according to the standard meteorological definition as follows: spring - March through May, summer - June through August, autumn - September through November, and winter - December through February. Following the convention of the local hydrologic office, April through September was defined as the flood season, while the remaining months were defined as the non-flood season.

Therefore, the Nanliu river basin (8600 km^2^) was selected as a case study to evaluate the applicability of TMPA products in small basin areas. This work aims to answer the following two questions: First, can TMPA products be used for a small basin area with the desired level of precision at all temporal scales (daily, monthly, seasonal, flood seasonal or non-flood seasonal and yearly)? Second, do the TMPA products drive hydrologic models well? The Nanliu River (Fig. 1), which flows to the sea, is the largest river in China’s Guangxi Zhuang Autonomous Region, with an approximate river length of 287 km and drainage area of 8600 km^2^. The basin is located within the longitude span from 109°30′E to 110°53′E and latitude span from 20°38′N to 23°07′N. Small dams are scattered throughout the basin to regulate natural hydrologic processes and may affect the simulation of the rainfall-runoff model. The Nanliu River is located south of the Tropic of Cancer and is in the subtropical monsoon climate zone of South Asia. The mean annual precipitation is approximately 1400–1760 mm and is mostly concentrated within the flood season (April to September). The average annual temperature range is 21.5 °C to 22.4 °C.

## Results and Discussion

### Spatial distribution of daily precipitation

**Figure 2:**
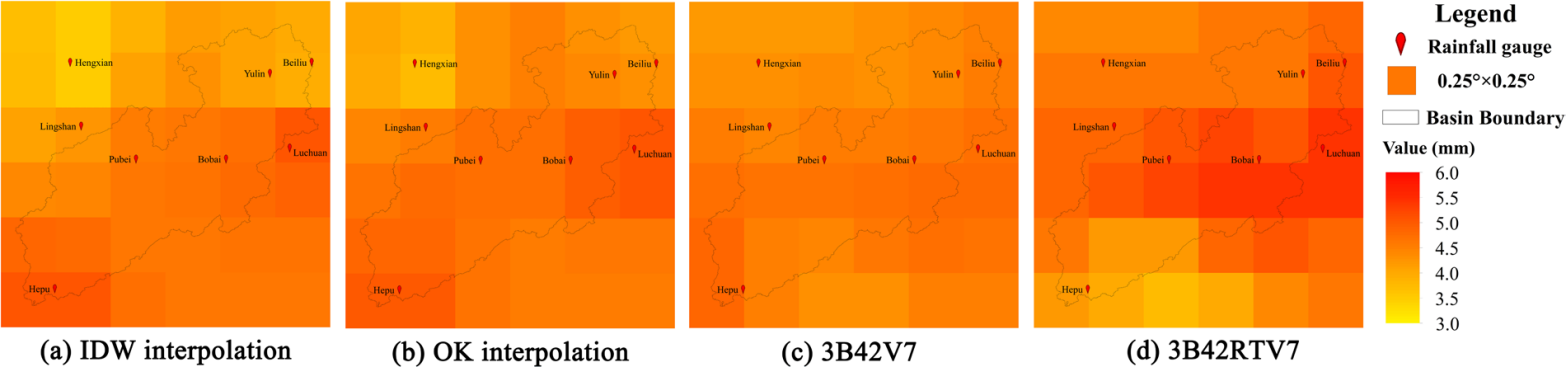
The spatial distribution of 9-year mean daily precipitation over the Nanliu river basin, derived from (**a**) IDW interpolation, (**b**) OK interpolation, (**c**) 3B42V7 and (**d**) 3B42RTV7. The maps were generated using ArcGIS10.1 (http://www.esrichina.com.cn/softwareproduct/ArcGIS/).

Figure 2 shows the spatial distribution of mean daily precipitation over the Nanliu river basin. The gauge-derived spatial distribution (Fig. 2(a),(b)) was derived from 9-year mean observation values from 8 gauge stations (7 reference stations selected for this study plus the Hengxian rainfall gauge located in the bounding rectangle of the Nanliu river basin) based on inverse distance weight (IDW) and ordinary kriging (OK) interpolation methods using ArcGIS 10.1, respectively. We averaged 9-year daily raster layers by using the Raster Calculator Module of ArcGIS and then generated the spatial distribution of TMPA products (Fig. 2(b),(c)). Similar spatial distributions of the gauge interpolation were found from IDW and OK interpolation methods. 3B42V7 has approximately the same spatial pattern as the gauge interpolation. 3B42RTV7 obviously overestimates the precipitation for the upper-middle basin and underestimates on the lower basin, whereas 3B43V7 shows better accordance with gauge interpolation, albeit with slight underestimation. The good performance of 3B42V7 is likely because 3B42V7 is a post-real-time product, adjusted from 3B42RTV7 by ground-based monthly GPCP/Climate Anomaly Monitoring System (CAMS) data. The improved performance of 3B42V7 compared to 3B42RTV7 is mainly attributed to the improved mean daily precipitation data, which decreases the bias.

In addition, we should realize that it would be difficult to quantify the uncertainties in interpolated gauge observations because daily precipitation occurs in a discrete pattern both in time and space and the interpolation of daily precipitation is very difficult.

### Temporal scale effect of precipitation datasets

**Figure 3:**
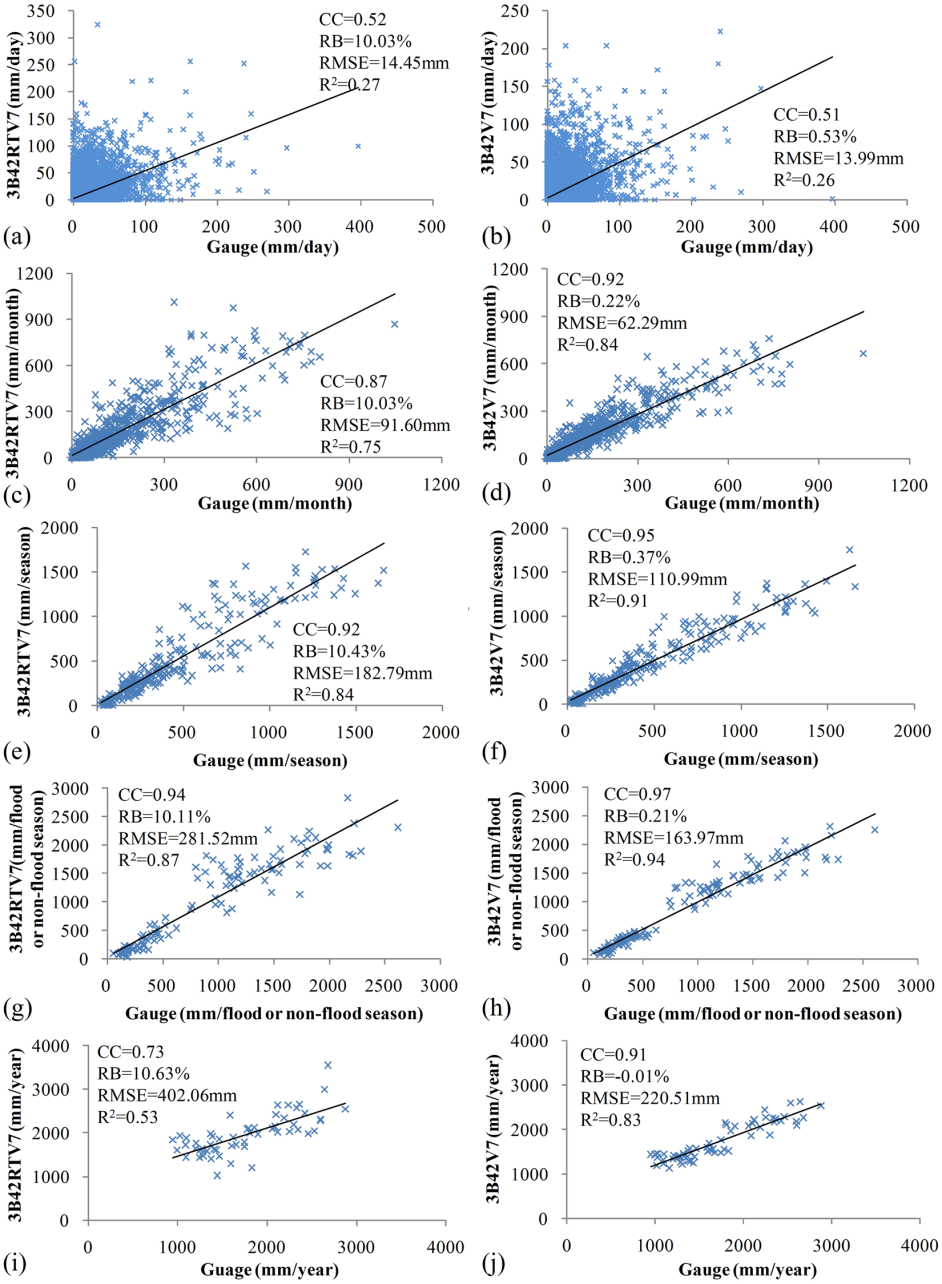
Scatter plots of grid-based daily, monthly, seasonal, flood seasonal or non-flood seasonal, and yearly precipitation at seven selected grid-boxes over the Nanliu river basin at relevant scales.

Precipitation data is typically used at different scales depending on the purpose. In this study, we built plots of 9-year daily, monthly, seasonal, flood-seasonal or non-flood-seasonal, and yearly comparisons of 3B42RTV7 and 3B42V7 data versus gauge observation data as shown in Fig. 3. Seven grid boxes were selected, each containing one gauge. With the relevant gauge observation values as references, correlation coefficient (CC), relative bias (RB), root-mean-square error (RMSE) and coefficient of determination (R^2^) were used to estimate the accuracy of TMPA products.

3B42RTV7 overestimates the precipitation by more than 10% (RB) at all temporal scales, which is in accordance with its performance with the spatial distribution of 9-year mean daily precipitation shown in Fig. 2(c). The data pairs between 3B42RTV7 and gauge observations were clustered more closely toward 3B42RTV7. In contrast, 3B42V7 only slightly overestimates the precipitation (RB: 0.53% at the daily scale, 0.22% at the monthly scale, 0.28% at the seasonal scale, 0.21% at the flood/non-flood seasonal scale, and −0.01% at the yearly scale). The same patterns were apparent for the RMSE values, i.e., that 3B42RTV7 exhibits greater RMSE than 3B42V7 in comparison with gauge observations (14.45 mm vs.13.99 mm at the daily scale, 91.60 mm vs. 62.29 mm at the monthly scale, 182.79 mm vs. 110.99 mm at the seasonal scale, 281.52 mm vs.163.97 mm at the flood/non-flood seasonal scale, and 402.06 mm vs. 220.51 mm at the yearly scale). Notably, the errors in 3B42RTV7 have larger increases over time than 3B42V7. This disparity is likely due to the effect of cumulated errors from the aggregation of daily precipitation errors.

Figure 3 clearly shows that longer averaging shifts the scatter diagram for both 3B42RTV7 and 3B42V7 toward the 1:1 line. At the same time, 3B42RTV7 has relatively poorer correlation than 3B42V7 with gauge observation data at all temporal scales except the daily scale. In general, 3B42V7 shows considerably better performance, with higher CC and R^2^ values. 3B42V7 is more consistent with gauge observations at all scales, as expected. Hence, this study demonstrates that 3B42V7 can be useful as an additional option for data sources.

**Figure 4:**
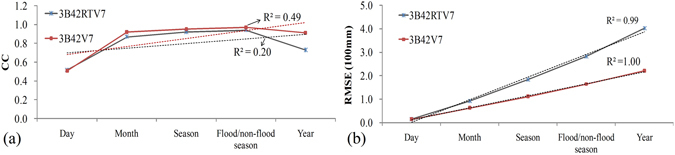
Temporal scale effect of TMPA products.

The temporal scale effect derived from the scatter plots shown Fig. 3 is depicted in Fig. 4 using CC and RMSE accuracy information. Clearly, the CC of the comparisons between TMPA products and gauge observations continues to increase up to the flood/non-flood seasonal scale, at which point it shows a relative decrease at the yearly scale (Fig. 4(a)). The greatest increases of the CC for both the 3B42RTV7 and 3B42V7 comparisons were at the monthly scale. The tendency of the CC for both 3B42RTV7 and 3B42V7 comparisons shows the same pattern. Similar change trends have been found in many places such as Hun-Tai basin in northeast China^8^, which also highlighted the notion that monthly gauges tend to be correlated over relatively large distances. The RMSE growth trends for both 3B42RTV7 and 3B42V7 comparisons are clear and linear (Fig. 4(b)). Both are statistically significant at the 95% confidence level. These results suggest that both TMPA products (3B42RTV7 and 3B42V7) have linear improvement in clustering and tend toward gauge observations with the accumulation of time.

### Detection capabilities of rain events and processes

**Figure 5:**
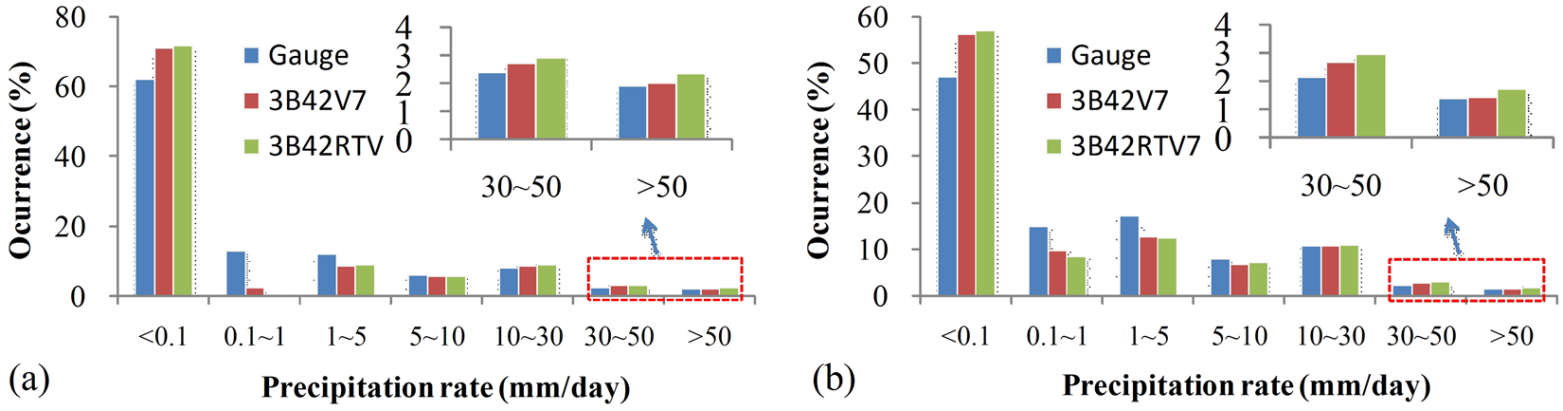
The probability distribution of the grid-based daily precipitation (**a**) and average daily precipitation (**b**) from the three precipitation products indicated.

Figure 5(a) and (b) show the probability distributions of daily precipitation for gauge observations, 3B42RTV7 and 3B42V7 at the grid scale and the basin scale, respectively. On one hand, at both spatial scales, TMPA products underestimate precipitation occurrence only when the precipitation rate is between 0.1 mm/day and 5 mm/day, which may imply that it is difficult for TRMM satellites to detect light rainfall. On the other hand, all TMPA products overestimate the occurrence of little or no rain at the grid and basin scales. Notably, all TMPA products show strong similarity with gauge observations for precipitation rates of 5–10 mm/day and 10–30 mm/day at both spatial scales. These findings suggest that 3B42V7 and 3B42RTV7 perform well for detecting precipitation at the 5–30 mm/day level. For precipitation rates greater than 30 mm/day (Fig. 5), 3B42V7 shows somewhat more frequent occurrence and 3B42RTV7 shows substantially more occurrence at both spatial scales. These results could explain why 3B42RTV7 overestimated precipitation by more than 10% at all temporal scales (Fig. 3).

**Figure 6:**
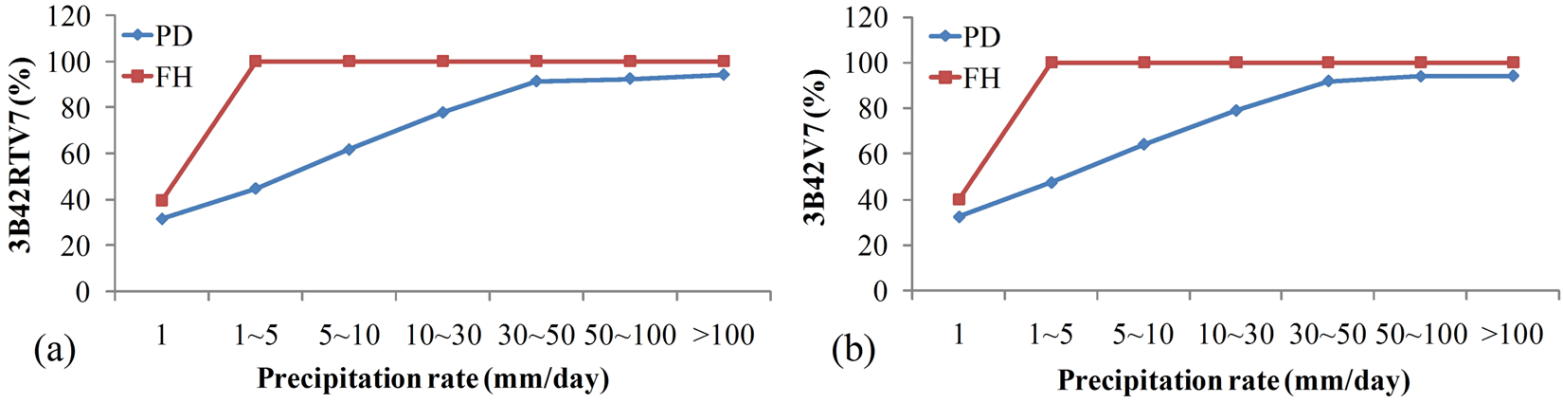
PD and FH for precipitation detection over the Nanliu river basin.

To estimate the precipitation detection capabilities of TMPA products, PD and FH were calculated as shown in Fig. 6 using equations (5) and (6). Several trends are apparent from Fig. 6. First, 3B42V7 and 3B42RTV7 have similar FH and PD trends and scores. FH values are as high as 100% when the precipitation rate is greater than 1 mm/day. Hence, all TMPA products have highly effective detection capabilities and capture all daily rain events with precipitation rates greater than 1 mm/day. The PD of 3B42V7 is generally better than that of 3B42RTV7. Notably, 3B42V7 and 3B42RTV7 captured approximately 90% of daily precipitation events when precipitation rates were greater than 30 mm/day. Obviously, both PD and FH of the TMPA products generally increased with the daily precipitation rate.

**Figure 7:**
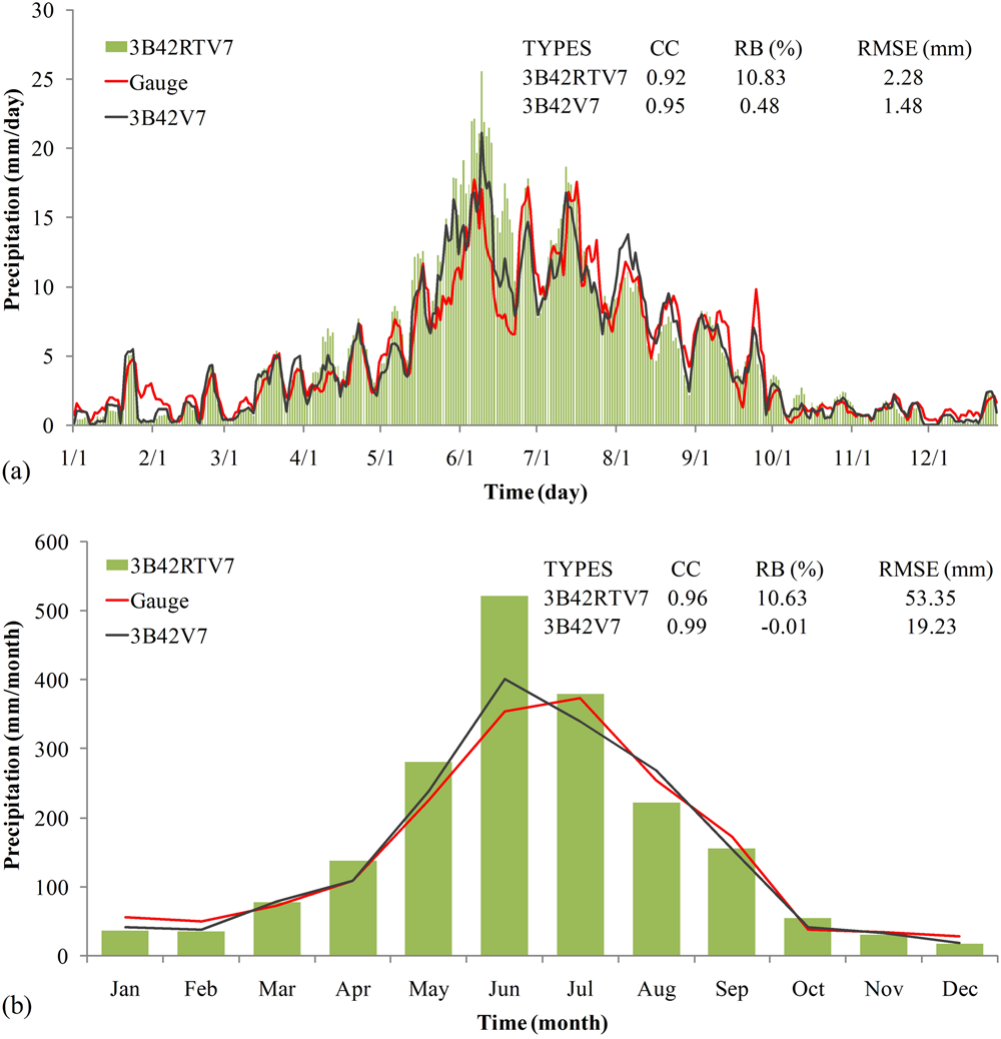
Selected grid-based mean daily (**a**) and monthly (**b**) precipitation over a year. A five-day moving average was used for daily precipitation for both gauge observations and TMPA products to enhance the trend patterns.

To estimate precipitation capture capabilities of TMPA products over an annual curve, annual cycle of precipitation was computed from 9-year mean daily and monthly precipitation data that were averaged for 7 gauges and 7 corresponding grid values of TMPA products (at the same locations as the 7 rainfall stations) at each day and month (Fig. 7). 3B42V7 and 3B42RTV7 adequately captured the pattern of the annual precipitation curve derived from gauge observations, including the peak and valley values. The value is a minimum in the winter, increases slowly during the spring, peaks in the summer, and is followed by a gentle decline during the autumn, as depicted by the curves in Fig. 7 showing high correlation (CC greater than 0.95). Notably, 3B42V7 matched well with gauge observations, as demonstrated by the small magnitude of RB (0.48% at the daily scale and −0.01% at the monthly scale).

### Hydrological evaluation of TMPA Products

**Figure 8:**
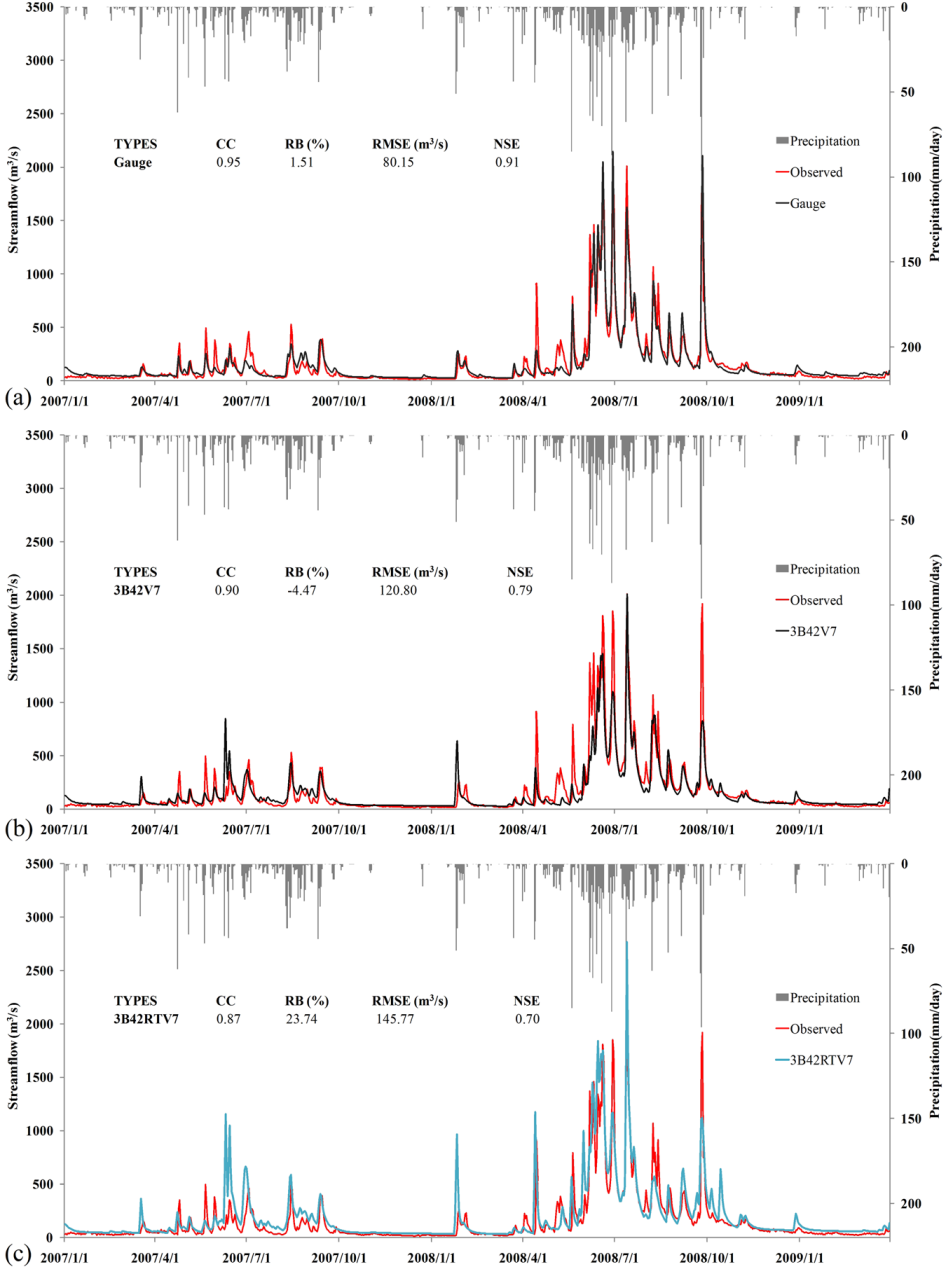
Daily simulation of Nanliu river discharge with inputs from either (**a**) gauge observations, (**b**) 3B42V7 or (**c**) 3B42RTV7. Rainfall inputs (gray bars) from gauge observations are plotted on the secondary ordinate. The red line represents the reference observations of river discharges from the Changle streamflow station.

For further assessment of the accuracy of TMPA products, hydrological simulations based on the products were conducted. We input daily 3B42V7, 3B42RTV7 and gauge observations into the Xin’anjiang model to compare stream flow observations as shown in Fig. 8. CC, RB (%) and NSE were used to evaluate the hydrological prediction capabilities of three datasets at the daily scale, and RMSE was adjusted accordingly. As shown in Fig. 8, gauge observation-based simulated stream flow shows greater consistency with the observed stream flow (as measured at the Changle hydrological station) than flows simulated based on 3B42V7 and 3B42RTV7, with a higher CC (0.95) and NSE (0.91) and a lower RB (1.51%) and RMSE (80.15 m^3^/s). The gauge observations-based simulation output captured most of the pattern of the observed flow curve, including 91% of total peak magnitudes or recessions and 100% of the total base flow. Similarly, the 3B42V7-based output also captured the pattern of the observed flow curve reasonably well, with high CC (0.90) and NSE (0.79) and low RB (−4.47%) and RMSE (120.80 m^3^/s), as evidenced by its capture of approximately 79% of total peak magnitudes or recessions and 100% of the total base flow. The high NSE value (0.79) means that the model is reliable. Compared to the gauge observations-based output, 3B42V7-based output was prone to larger deviations from the observed hydrography. In general, 3B42V7 showed good performance when used for simulating a hydrologic model. This implies that 3B42V7 can meet the required level of precision and has good applicability as an additional option for water management studies. In contrast, the 3B42RTV7-based output showed a poorer hydrological prediction value than that based on 3B42V7, with the lowest CC (0.87) and NSE (0.7) values; similar findings have also been demonstrated for the Ganjiang Basin region^3^. The 3B42RTV7-based output heavily overestimated the stream flow by approximately 24% (RB = 23.74%). Perhaps the high bias of daily 3B42RTV7 (RB = 10.03%) was magnified through the hydrological model, making a poor 3B42RTV7-based result. The same situation may occur in daily 3B42V7.

## Conclusion

Two TMPA products (3B42V7 and 3B42RTV7) were evaluated against data from seven gauge observations for a 9-year period at different scales over the Nanliu river basin. In addition, the TMPA products and gauge observations were input into the Xin’anjiang model to evaluate the capabilities of these products to accurately drive hydrologic models. In this study, we found that

- The bias-adjusted product 3B42V7 exhibits greater accuracy at temporal and spatial scales compared to the unadjusted product 3B42RTV7. 3B42RTV7 overestimates precipitation by more than 10% at all scales. In contrast, 3B42V7 overestimates precipitation only slightly (0.53% at the daily scale, 0.22% at the monthly scale, 0.37% at the seasonal scale, 0.21% at the flood/no-flood seasonal scale and −0.01% at the yearly scale). In addition, 3B42V7 agrees well with gauge observations, with CC values mostly exceeding 0.9 except for the value of 0.51 at the daily scale. Correspondingly, 3B42RTV7 comparisons have larger RMSEs than 3B42V7 comparisons against gauge observations (14.45 mm vs. 13.99 mm at the daily scale, 91.60 mm vs. 62.29 mm at the monthly scale, 182.79 mm vs. 110.99 mm at the seasonal scale, 281.52 mm vs. 163.97 mm at the flood/no-flood seasonal scale and 402.06 mm vs. 220.51 mm at the yearly scale).
- The CC trends for 3B42RTV7 and 3B42V7 comparisons increase with increasing time scales. The maximum CC improvement points for 3B42RTV7 and 3B42V7 comparisons occurred from the daily scale to the monthly scale.
- 3B42V7 is better than 3B42RTV7 for the detection of daily precipitation. In terms of PD values, 3B42V7 is generally better than 3B42RTV7 at all daily precipitation levels.
- 3B42V7 is more accurate than 3B42RTV7 at hydrologic flow prediction, with higher CC (0.90 vs.0.87) and NSE (0.79 vs.0.70) and lower RB (−4.47% vs. 23.74%) values compared to actual flow data. 3B42V7 adequately captured the pattern of the observed flow curve.

Overall, 3B42V7 and 3B42RTV7 both showed good performance in terms of precision and in hydrological simulation. However, comparing the two products, 3B42V7 is an upgraded version of 3B42RTV7 that shows good precipitation detection accuracy and improved hydrologic prediction capability. 3B42V7 can therefore be treated as a suitable data source for application in a small basin case.

## Methodology

### Interpolation methods

A number of modern interpolation methods have been proposed for precipitation such as IDW, ordinary kriging (OK), radial basis function, local polynomials, nearest neighbor, linear regression, and geographically weighted regression and artificial neural network (ANN). Many papers have been dedicated to the comparison of these interpolation methods, but it is hard to set up a relative international notion of which method is better. In contrast, IDW and OK methods are most commonly used in interpolation of climate variables. We consulted other researchers^3, 15^ in China, and found that IDW and OK were primarily used as spatial interpolation methods.

### Statistical Metrics

The accuracy of precipitation products is the primary issue for their application in areas of research, including climate change, hydrological simulation, etc. Therefore, it is necessary and useful to evaluate the performance of 3B42RTV7 and 3B42V7 against reference gauge observations. The assessments were performed by direct comparison of 3B42RTV7 and 3B42V7 with gauge observation data at several reference scales. The statistical tools of CC, RB, RMSE and R^2^ were used to assess accuracy. These three indexes were defined as follows:

1 $$CC=\frac{{\sum }_{{\rm{i}}=1}^{{\rm{n}}}({{\rm{QPE}}}_{{\rm{i}}}-\bar{{\rm{QPE}}})({{\rm{gauge}}}_{{\rm{i}}}-\bar{{\rm{gauge}}})}{\sqrt{{\sum }_{{\rm{i}}=1}^{{\rm{n}}}{({{\rm{QPE}}}_{{\rm{i}}}-\bar{{\rm{QPE}}})}^{2}{\sum }_{{\rm{i}}=1}^{{\rm{n}}}{({{\rm{gauge}}}_{{\rm{i}}}-\bar{{\rm{gauge}}})}^{2}}}$$

2 $$RB=\frac{{\sum }_{{\rm{i}}=1}^{{\rm{n}}}({{\rm{QPE}}}_{{\rm{i}}}-{{\rm{gauge}}}_{{\rm{i}}})}{{\sum }_{{\rm{i}}=1}^{{\rm{n}}}{\rm{gauge}}}\times 100 \% $$

3 $$RMSE=\sqrt{\frac{{\sum }_{{\rm{i}}=1}^{{\rm{n}}}{({{\rm{QPE}}}_{{\rm{i}}}-{{\rm{gauge}}}_{{\rm{i}}})}^{2}}{{\rm{n}}}}$$

4 $${R}^{2}={\rm{CC}}\times {\rm{CC}}$$

where QPE is the precipitation value from a TRMM product; gauge is the value from gauge observation data; $\overline{{\rm{QPE}}}\,$and $\overline{{\rm{gauge}}}$ are mean values. n is record number; CC is the linear correlation between a TRMM product and the reference and is dimensionless; RB is the relative difference between a TRMM product and the reference and is given as a percentage difference; RMSE is the error between TRMM products and reference and is in units of mm/time. The range of values allowed for the CC is 0 to 1. Perfect values of CC, RB and RMSE are 1, 0 and 0, respectively.

To evaluate the detection capabilities of TRMM precipitation products relative to gauge observation at the daily scale, Probability of Detection (PD) and Frequency of Hit (FH) were used. High PD values indicate that many precipitation events were detected by TRMM products, and high FH values indicate that many precipitation reports from TRMM products were accurate^16^. PD and FH are calculated using the following equations:

5 $$PD=\frac{{n}_{11}}{({n}_{11}+{n}_{01})}\times 100 \% $$

6 $$FH=\frac{{n}_{11}}{{n}_{11}+{n}_{10}}\times 100 \% $$

where the n_QPE,gauge_ value is 0 or 1, and its first subscript “QPE” and second subscript “gauge” represent the state of precipitation events from TRMM products and gauge observations, respectively, at the daily scale. Here, 1 indicates that the precipitation rate is ≥0.1 mm, whereas 0 indicates that it is not. Accordingly, n_11_ indicates that the TRMM products and gauges both reported a rain event; n_10_ indicates only the TRMM products reported a rain event; n_01_ indicates that only the gauges reported a rain event; and n_00_ indicates that neither the TRMM products nor the gauges reported a rain event.

### Hydrological model

A hydrological model is an important tool for hydrological simulation. The Xin’anjiang representative conceptual model has been widely used in humid and semi-humid areas of China and around the world since its development in the 1970s. The main feature of the Xin’anjiang model is the concept of runoff formation upon repletion of storage, i.e., that runoff is not produced until soil moisture content of the aeration zone, that is a zone between the water table and the surface, reaches field capacity, and thereafter runoff equals rainfall excess without further loss^3, 17^. Three runoff components (surface runoff, subsurface runoff and groundwater) and three evapotranspiration layers are packaged into the Xin’anjiang model. The successful application of the Xin’anjiang model depends primarily on how well the model is calibrated. The auto-calibration method is widely applied in parameter optimization because it is more efficient and effective and can avoid interference from anthropogenic factors^18^. Hence, the Shuffled Complex Evolution-University of Arizona (SCE-UA) algorithm^19^ was used in this study to calibrate 15 major parameters for the Xin’anjiang model for data from January 2007 to March 2009 at the daily scale before hydrologic simulation. The definitions and calibrated values of the 15 parameters are given in Table 1. We assumed that the impacts of dams and human regulation on the simulated Nanliu river flow were considered in the auto-calibration process.

**Table 1:** Calibrated parameters and calibrated values for the Nanliu river basin.

No.	Parameters/units	Definition	Calibrated value
1	K (dimensionless quantity)	Pan evaporation coefficient	0.880
2	C (dimensionless quantity)	Evapotranspiration coefficient from deep layer	0.175
3	WUM (mm)	Tension water capacity from upper layer	82.660
4	WLM (mm)	Tension water capacity from lower layer	149.310
5	WDM (mm)	Tension water capacity from deep layer	266.385
6	B (dimensionless quantity)	Exponential number of storage capacity distribution curve	0.594
7	SM (mm)	Areal mean free water storage capacity	54.070
8	EX (dimensionless quantity)	Parameter in the distribution of free water storage capacity	0.935
9	KG (dimensionless quantity)	Outflow coefficient of the ground water from free water	0.254
10	KSS (dimensionless quantity)	Outflow coefficient of the interflow from free water	0.499
11	KKG (dimensionless quantity)	Groundwater recession coefficient	0.997
12	KKSS (dimensionless quantity)	Interflow recession coefficient	0.841

The Nash-Sutcliffe efficiency coefficient (NSE) is typically used to assess the efficiency of hydrology models. NSE is calculated using the following equation:

7 $${\rm{NSE}}=1-\frac{{\sum }_{{\rm{t}}=1}^{{\rm{T}}}{({{\rm{Q}}}_{{\rm{o}}}^{{\rm{t}}}-{{\rm{Q}}}_{{\rm{m}}}^{{\rm{t}}})}^{2}}{{\sum }_{{\rm{t}}=1}^{{\rm{T}}}{({{\rm{Q}}}_{{\rm{o}}}^{{\rm{t}}}-\mathop{\overline{{{\rm{Q}}}_{{\rm{o}}}}}\limits^{\cdot })}^{2}}$$

where t represents time point; Q_o_ represents observed values from the hydrologic station; $\bar{{Q}_{{\rm{o}}}}$ is average Q_o_; and Q_m_ represents the simulated values from the hydrology models. The perfect value of NSE is 1, indicating that the model is credible. An NSE value close to 0 indicates that the simulation results are approximately the same as the average of observed values, which indicates that the model is generally credible, although with large errors in the process.
